# Mindfulness-Based Programs Improve Psychological Flexibility, Mental Health, Well-Being, and Time Management in Academics

**DOI:** 10.3390/ejihpe10040073

**Published:** 2020-11-03

**Authors:** Gabriel A. B. Marais, Sophie Lantheaume, Robin Fiault, Rebecca Shankland

**Affiliations:** 1 LIP/PC2S-EA 4145, Université Grenoble Alpes, 38000 Grenoble, France; lantheaume.sophie@hotmail.fr (S.L.); rebecca.shankland@univ-grenoble-alpes.fr (R.S.); 2 LBBE-UMR 5558, CNRS/Université Claude Bernard Lyon 1, 69622 Villeurbanne, France; 3 LEAF, Instituto Superior de Agronomia, Universidade de Lisboa, 1349-017 Lisboa, Portugal; 4 Hôpital Privé Drôme Ardèche, 07500 Guilherand-Granges, France; 5 Ecole des Psychologues Praticiens, 69003 Lyon, France; robin.fiault@gmail.com

**Keywords:** occupational stress, time management, well-being, mindfulness, academia

## Abstract

(1) Background: Occupational stress is high in academia, and is partly related to time pressure. Mindfulness-based programs are known to be effective in reducing stress and increasing well-being. Recent work suggested that these programs may also improve time management. This study tested the effects of a mindfulness-based program on academics’ psychological flexibility, mental health, well-being, and time management. (2) Methods: The study was conducted in a French research department. Participants were offered to join a mindfulness-based program (n = 21) or to be on a wait-list control group (n = 22). Self-reported measures of psychological flexibility, mental health (stress, anxiety, and depression symptoms), well-being, and time use were collected before and after the eight week program. (3) Results: Results showed that psychological flexibility, mental health, well-being, and efficient time use significantly increased in the intervention group compared to the control condition. (4) Conclusions: The results suggested that the mindfulness-based programs were effective in improving adaptive functioning, well-being, and optimal time use in academia, thus underlining potential useful perspectives to help academics improve mental health and time management.

## 1. Introduction

About one third of workers experience mental health issues such as chronic stress in developed countries, which results in significant human and financial costs [1,2]. Almost all sectors are affected and academia is no exception. Surveys in UK, Australia, Canada, and other countries have revealed high to very high levels of occupational stress in academia, a situation that is shared by all disciplines [3,4,5,6,7,8,9,10]. Occupational stress seems even higher in academics compared to the general population or other similar “white-collar” (office) workers [3,10]. Identified stressors in academia are numerous [3,10,11] and include cuts in funding and resources (e.g., [12]), job insecurity (e.g., [4,12]), pressure to publish and to obtain external funding (e.g., [4,12,13]), increased student/staff ratio (e.g., [12,13,14,15]), increased workloads (e.g., [4,12,13]), working outside office hours (e.g., [13]), work-life conflicts (e.g., [9]), slow career advancement (e.g., [4,12]), lack of recognition (e.g., [12]), poor management practices (e.g., [12,13]), and lack of trust in institutions (e.g., [12]). Exposure to stressors affects both the mental and physical health of the academics (e.g., difficulties concentrating and making decisions, decreased self-esteem, depression, sleep disturbances, headaches, stomachaches, susceptibility to infections), and also has organizational consequences (e.g., job dissatisfaction, decreased productivity, teaching below standard, decreased level of organizational commitment, seeking jobs elsewhere [3,9,12,16,17]). In France, few data are available, but they suggest occupational stress is very high (with about half of the academics reporting high level of stress), potentially triggered by heavy workloads, work-life conflicts, and poor management practices [18]. Occupational stress results from individuals’ inability to cope with the pressures of their job, and time pressure in particular [19,20]. Academics often experience a strong disparity between the time necessary to perform their activities and the actual time available, which results in time stress [21]. Consequently, academics’ worktime is longer than expected, 45–50 hours a week on average, as found in an international survey [22]. Teaching and administrative duties put the highest constraints on academics’ time schedule, often at the cost of research [23]. It is very common for academics to work outside working hours in the evenings, on the weekends, and during holidays, and to show up at work even when sick [24]. The culture of overwork is deeply rooted in academia [21]. Time stress not only decreases well-being but also performance (teaching and research) of academics. Therefore, challenging the culture of speed in academia becomes necessary [21,25].

In France, time stress is probably very common in academia, although quantitative data are lacking [26]. Most of the French academics are lecturers and professors (which implies that they are not only researchers but also teachers) hired by the university. According to their official status, they have two missions: research and teaching, with 192 hours of annual face-to-face teaching. In a 2009 decree, these missions were subdivided into eight different tasks, without mentioning administration explicitly. A total freedom and autonomy to conduct these missions is stated. CNRS (Centre National de Recherche Scientifique, which is the national research institute in France) investigators, the other main type of French academics (mainly research-focused), have 17 items (different missions) to fill in their annual evaluation. Multi-activity and autonomy are thus the pillars of time management in academia. Complaints about time management from academics can appear paradoxical, as one may think that with freedom and autonomy, time adjustments are easy [26]. This paradox is in fact shared by all academics worldwide [25]. The corporatization of the university that has affected academia in France and elsewhere explains this paradox [21,25,26]. A combination of high pressure from the university (demands for productivity at a frantic pace and heavy administrative duties) and autonomy results in highly blurred boundaries between work and life, highly chaotic time schedules, and the co-existence of multiple academic cycles and timeframes (teaching, internships, grant proposals, etc.). Different tactics are developed by academics to adjust: self-constrained time allocation for the different activities, self-imposed rules of not working at home, setting up routines, alternating high and low concentration-required activities, and avoiding, minimizing, or postponing some activities [26]. Time management in academia is thus highly complex and is time consuming in itself, something academics are not always aware of [26]. Based on these observations, our research question was: is it possible to help academics increase efficient time use at work and reduce their levels of stress? The aim of this pilot study was to test the efficacy of a mindfulness-based program compared to a wait-list control condition on these dimensions. 

### 1.1. Mindfulness-Based Interventions to Reduce Occupational Stress

Research suggests that workload and occupational stress have been increasing over time in academia [27,28], despite some organizational responses to the problem implemented by certain universities [28]. There is thus clearly a need for both rethinking the system and finding some novel practical solutions. As mindfulness-based programs have been shown to reduce stress in various contexts [29,30], this may represent a means of responding to this problem, and a growing number of institutions worldwide have begun to implement such programs [31]. Mindfulness can be defined as the ability to focus attention on present moment experience in order to be aware of sensations, thoughts, and feelings, and to observe with curiosity and a non-judgmental attitude, without reacting immediately to inner experience (whether it maybe a thought, a negative affect, or a tension in the body). Mindfulness practices, such as breathing or body awareness, mindful walking, or mindful eating, are considered as a means of cultivating this ability [32]. Mindfulness is widely studied in psychology, neurobiology, and medical research (more than 7000 publications in PubMed, the main database of biology and medical research), and has been developed in organizational contexts in order to reduce occupational stress. Indeed, a survey carried out in 2016 in the USA reported that 13% of the employees were involved in mindfulness-based programs in the USA [31]. These programs have been shown to reduce stress, anxiety, depression, burnout risk, and to increase perceived quality of life [29]. Hence, they both reduce negative affect [29] and increase positive affects [33]. Mindfulness-based programs have significant effects in highly stressed populations such as medical doctors or university students, but also in the general population [34]. Studies show that the effects of mindfulness-based programs on stress can be captured by self-reported questionnaires as well as biological measures (e.g., stress hormone blood concentration, epigenetic markers, see [31,35,36,37]). 

In organizational settings, mindfulness-based programs have shown positive outcomes in terms of stress reduction [38], reduced emotional exhaustion and turnover intentions [39], reduced burnout [40], as well as well-being promotion (e.g., [41]). Mindfulness-based programs at work have also been shown to help increase concentration, cognitive abilities such as attentional control and cognitive flexibility, as well as emotion regulation and behavioral flexibility (reducing automatic behaviors triggered by stressful situations, for example), and have also been shown to increase performance at work and inter-personal relationships (e.g., [31]). Past research has shown that human beings spend almost 50% of their time mind wandering, which negatively affects their well-being and performance at work [42]. Mindfulness trainees experience less mind-wandering and focus on a task with more ease [31]. Mindfulness has also been shown to help developing social skills such as empathy and kindness, and reacting in an adapted way, even when facing relationship tensions. Through mindfulness practices in organizational settings, communication, inter-personal relationships, work environment, and quality of life at work can be improved [31]. 

### 1.2. Mindfulness-Based Interventions and Time Management

Mindfulness has been suggested to help relate to time differently, with a focus on relating to the present moment rather than continuously trying to anticipate the future or ruminate past events [32]. Mindfulness-based programs may therefore be considered as a means of acting upon time use and management. The theory of time perspectives posits that there are six temporal frames: past positive (PP, warm, positive and nostalgic view of the past), past negative (PN, negative and aversive view of the past), present hedonic (PH, a live-for-the-moment attitude involving immediate pleasure seeking), present fatalistic (PF, hopeless and helpless attitude toward the present), future negative (FN, negative views or attitudes toward the future), and future positive (FP, future orientation involving optimism, planning, and striving for future rewards, see [43,44]). A balanced time perspective (BTP, high to moderate PP, PH, and FP and low PN, PF, and FN) is correlated with low levels of stress, anxiety, and depression and high levels of well-being and satisfaction with life [43,44,45,46]. Trait-mindfulness has been shown to correlate positively with measures of BTP [47,48,49,50,51,52]. Furthermore, BTP is not a fixed trait and can be changed (although not completely) through mental training, and it has been found that mindfulness-based programs can increase BTP and well-being [49,53,54]. 

The situation of academics with high occupational stress and strong time pressure and calls for slowing down academic life (i.e., “the slow professor”, see [21]) suggest mindfulness could be particularly recommended in this context [55]. Mindfulness-based programs could improve academics’ mental health as well as time management [56]. Mindfulness could help academics focus on the task they are doing and reduce the time to perform the task by increasing attentional resources, which can be otherwise engaged in the inhibition of task-irrelevant thinking potentially caused by anxiety. Academics’ multi-activity imposes a high rate of task switching during the day. Going from one mode (e.g., lecture mode) to another (e.g., meeting mode or research mode) may be challenging. It can result in procrastination or lack of concentration, which reduce efficacy [56]. Mindfulness could ease the process of concentrating on a new task while reducing mind wandering, which is linked to other ongoing projects. The effect of mindfulness on attention switching–defined as the ability to shift back and forth between multiple tasks, operations, or cognitive strategies—may however be complex [57]. Other possible benefits include finding better solutions in case of time conflicts [56]. Most importantly, mindfulness could be a means of reducing perceived emergency, which leads from one task to another while sometimes losing track of one’s life goals and priorities. Research has shown that mindfulness-based programs can enhance psychological flexibility, which is defined as the ability to act in line with one’s values and life goals rather than reacting to situations while missing the broader perspective [58]. Psychological flexibility refers to the ability to choose the most appropriate behaviors to serve one’s life goals and values. It implies six processes: acceptance (rather than trying to avoid emotions or thoughts), cognitive defusion (rather than considering one’s thoughts as truths), present moment awareness (being aware of one’s current state, sensations, emotions), self as context (being able to observe one’s thoughts and emotions from a broader perspective), clarity of values (knowledge of one’s values), and engagement in actions related to these values. Increasing psychological flexibility through mindfulness-based programs may thus be a means of reducing pressure on academics by helping them to take perspective and remain connected to their values, their needs, and their current state. Conversely, psychological inflexibility has been shown to be correlated to anxiety and depression symptoms (for a review, see [59]). In the same way, lack of psychological flexibility explained between 16% and 28% of the variance observed in a variety of mental health indicators used in research studies [60]. Luckily, psychological flexibility may be cultivated through psychological interventions and notably through mindfulness-based programs. For example, a mindfulness-based program has been shown to enhance psychological flexibility, thereby reducing burnout in medical staff [61]. Furthermore, this ability to choose behaviors that are most appropriate to attain one’s goals may be helpful to increase effective time management. 

This study therefore aimed at further analyzing the effects of a mindfulness-based program on psychological flexibility, mental health, and time management in a French academic setting. The hypothesis was that participating in a mindfulness-based program would enhance psychological flexibility, mental health, well-being, and time management compared to a wait-list control group.

## 2. Materials and Methods 

The study was conducted in a biology research department and included university lecturers and professors, investigators, technicians, administrative staff, PhD students, and postdoctoral students. This department is one of the largest of that University, which is one of the largest French Universities with about 40,000 students. The research department includes permanent staff (academics, technicians and administrative staff) representing 124 individuals, short-term contract employees (postdoctoral students, PhD students, technicians and invited scientists) representing 60 researchers, and interns (Masters interns mainly) representing up to 100 students. 

A total of 43 out of the 184 staff members took part in the study, with 21 in the intervention group and 22 in the control group. Two participants did not reply to the online questionnaire at t2 (post-intervention) and were not included in the analyses. Thus, the data of 41 participants were used to perform the statistical analyses. Intervention and control groups were comparable in terms of gender, age, and type of contract (see Table 1). Compared to the total research department population, the studied sample was female-biased (~60% versus 46%) and permanent staff was overrepresented (~75% versus 68%, see Table 1). Participants were slightly younger than the mean age in this department.

The study was carried out in accordance with the 1964 Helsinki declaration and its later amendments. Informed consent was obtained from all participants included in the study. In October 2017, a short email of about 300 words was sent to all department members. This email briefly explained the project (context, objectives, timeline). The members who were interested to take part in the study could sign up in an online shared-document (using Framadate), where they were asked to indicate whether they were willing to join the first intervention group or the wait-list control group if the time of the sessions during the week did not fit with their schedule. Furthermore, by giving the participants the choice to start the program immediately or later, it also enabled the confirmation that the level of motivation to engage in such practices was sufficient. The intervention took place in late 2017–early 2018; t2 data were collected in spring 2018. The data analyses and investigation took place in late 2018–early 2019. The manuscript was written late 2019–early 2020. 

The only condition to participate to the control group was not to join an independent mindfulness-based program between t1 and t2. The vast majority of participants had no previous mindfulness experience. Gifts (a book on positive psychology or mindfulness) were offered to the control group participants to encourage and thank them for completing the online questionnaires. Informed consent was given by the participants at the beginning of the online questionnaire. This questionnaire was developed using Limesurvey (https://www.limesurvey.org), a professional tool for online surveys, in which anonymity is guaranteed. The “automatically generated token” mode, which guarantees that each participant has a unique and confidential access to the questionnaire, was used. 

The mindfulness-based program was adapted by a psychologist, a MBCT (Mindfulness-Based Cognitive Therapy; [62]) instructor, specialized in organizational psychology, who had already given various stress reduction and well-being programs in this university. The themes and mindfulness practices of the eight sessions were based on the classical MBSR and MBCT programs while adapting the examples and some psychoeducational aspects to organizational settings. The sessions are comprehensively described in Table 2. The whole program was designed to help the participants manage their stress at work and their relation to time-management. Each session lasted two hours, and began with a short meditation called “what do I come with today?”, lasting five to 10 min. Then, participants were guided through a practice, such as a body scan, for 30 to 45 min. After this guided practice, participants were invited to share their experience since their last session: How did they meditate at home and at work? What did they experience? What differences did they observe in their everyday experience in relation to the program? After this 30-minute discussion, another exercise was proposed, such as mindful walking for 15 min. After each exercise, participants were invited to describe their experience, reporting their sensations, feelings, and thoughts. At the end of the session, some practices were proposed for their everyday practice until the next session. For example, it was suggested that they experience breathing meditation 15 min daily using an audio file or a smartphone application. Last, participants were given one “memo sheet” per session, summarizing the different steps they went through. These memo sheets were supports for the participants along the program: they could refer to the memo sheet between sessions or after the end of the program. 

In order to plan the sessions, participants of the intervention group were asked their availabilities using a doodle poll. The 16 slots of two hours with the maximum number of available participants were used to maximize participation. Participants were split in two subgroups of 10 participants. Participation rate over the whole program was 80.4%.

The total online questionnaire was composed of 86 items. Tests by beta-testers showed that the questionnaire could be filled in 15–20 min. The questionnaires used in this study are detailed in Table 3 along with their internal reliability.

Psychological flexibility was measured using the Multidimensional Psychological Flexibility Inventory [63] aimed at capturing the six dimensions of psychological flexibility, a concept from Acceptance and Commitment Therapy (ACT, see [58]): *acceptance* (i.e., willingness to contact unwanted experiences fully), *contact with the present moment* (i.e., being in touch and aware of one’s experiences), *self as context* (i.e., keeping perspective of oneself within one’s experiences), *defusion* (i.e., being able to step back from unwanted experiences without getting stuck in them), *committed action* (i.e., maintaining behaviors that move toward important aspects of life), and *values* (i.e., staying connected to the areas of life that are important, giving direction to behaviors). MPFI score increases with ACT and mindfulness training [63]. MPFI score was computed using the instruction manual for users.

Depression, anxiety, and stress were measured using the DASS-21 (Depression, Anxiety, Stress Scale, [64]), which measures the frequency of symptoms (e.g., dryness in the mouth, no positive feelings, difficulties breathing, etc.). From the raw answers to the DASS-21, we computed the stress, anxiety, and depression scores using DASS-21 developer guidelines and the relevant items. Scores were multiplied by two and scores between 0–9 (DASS-21-D), 0–7 (DASS-21-A) and 0–4 (DASS-21-S) were considered normal, scores between 10–13 (DASS-21-D), 8–9 (DASS-21-A) and 15–18 (DASS-21-S) were considered mild, scores between 14-20 (DASS-21-D), 10–14 (DASS-21-A) and 19–25 (DASS-21-S) were considered moderate, scores between 21–27 (DASS-21-D), 15–19 (DASS-21-A) and 26–33 (DASS-21-S) were considered severe, and scores 28 (DASS-21-D), >20 (DASS-21-A), and >34 (DASS-21-S) were considered extremely severe accordingly to the developer guidelines.

Well-being was measured using the Warwick–Edinburgh Mental Well-Being Scale (WEMWBS, [65]), which is a one-dimensional scale that measures subjective (i.e., positive emotions) and psychological well-being (i.e., self-acceptance, personal growth, meaning in life, etc.) dimensions.

Optimal time use, which was defined, based on the literature review and combining different models by [66], as satisfying (due to having a healthy balance between areas of life and choosing self-congruent activities) and effective (spending time efficiently and experiencing a sense of mastery over the time one has), was measured by the Optimal Time Use Inventory (OTUI) developed by these authors, which captures the different dimensions of optimal time use: self-congruence of daily activities (an experience of alignment between the tasks and activities one typically does in everyday life and one’s values, goals, and priorities), balance between daily activities directed towards different life goals and domains (satisfaction with the way one distributes time and resources across different activities), efficiency of time use (absence of procrastination and of time experienced as wasted in one’s daily life, which requires organization and planning of activities, initiation and execution, motivation), control over time or time mastery (absence of time anxiety and time pressure experienced on a daily basis, which requires a responsible attitude towards one’s plans and commitments as well as the ability to align them with realistic time limits). We computed the global OTUI score and a score for each of the OTUI dimension (self-congruence, balance, efficiency, and control) following Osin and Boniwell’s recommendations.

Although answers were anonymous, data collected at t1 and t2 on the same participant were paired using a secret code that participants were asked to generate and keep with them. Data pairing was double-checked by making sure that demographic information for any given participant at t1 and t2 matched.

All statistical analyses were performed using the Statistical Package for Social Sciences (SPSS.22) software. Normal distribution and homoscedasticity of all the variables was checked and confirmed, which allowed us to use standard parametric statistical tests. The effects of the independent variable (group) on the dependent variables (psychological flexibility, stress, anxiety, depression, mental well-being, optimal time use, self-congruence, balance, efficiency, and control) were analyzed using repeated measures analysis of variance (ANOVA). The threshold of significance was *p* < 0.05, but marginally significant effects (*p* < 0.1) were also reported. Effect sizes are presented using partial eta-squares (η^2^). This value indicates the proportion of specific variance explained by the factor when the effect of the other factor is controlled. Effect sizes are considered as follows: small (0.01 ≤ η^2^ ≤ 0.05), medium (0.06 ≤ η^2^ ≤ 0.13), and large (η^2^ ≥ 0.14) [69].

## 3. Results

Baseline data showed that before the program, intervention and control groups were not identical in terms of mean psychological flexibility, DASS-21 scores, well-being index, and OTUI. The intervention group had significantly less psychological flexibility, well-being, and optimal time use than the control group. Anxiety and depression were marginally higher and self-congruent time use marginally lower in the intervention group than in the control group (see Table 4). These data suggest that participants who joined the intervention group first tended to have a lower level of mental health and optimal functioning (psychological flexibility and time management).

It is useful to note that in this sample, many participants were stressed, with 75.6% reporting mild to extremely severe stress, 29.1% reporting anxiety symptoms, and 51.2% reporting depressive symptoms (see Table 5).

### 3.1. Effects of the Intervention on Psychological Flexibility, Mental Health and Well-Being

**Figure 1:**
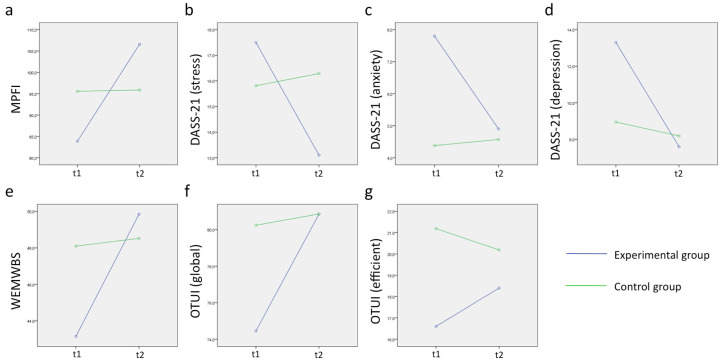
(**a**) Psychological flexibility, (**b**–**d**) mental health, (**e**) well-being, and (**f**,**g**) time use before (t1) and after intervention (t2) for intervention and control groups. Only variables with marginally significant or significant p-values are shown.

In order to test the effect of the program on psychological flexibility, mental health, and well-being, we ran a mixed repeated measures ANOVA considering both time (t1, t2) and group (intervention, control). We focused on the interaction term of this ANOVA between time and group. The time*group interaction showed a change in one group (and not the other) between time t1 and t2 (see Table 6). Psychological flexibility significantly increased in the intervention group between t1 and t2, and mental health scores (DASS-21) were reduced between t1 and t2 in the intervention group, although this was significant only for the depression score, while changes were marginally significant for stress and anxiety scores (Table 6 and Figure 1). The well-being score (WEMWBS) significantly improved in the intervention group between t1 and t2 (Table 6 and Figure 1).

### 3.2. Effects of the Intervention on Time Use

ANOVAs showed that the global OTUI score and the efficient time use OTUI score changed specifically for the intervention group between t1 and t2, with marginally significant and significant p-values respectively (Table 6 and Figure 1).

## 4. Discussion

The aim of this study was to analyze the effect of a mindfulness-based program on psychological flexibility, mental health, well-being, and optimal time management in academics. Compared to the wait-list control condition, the intervention group showed improved psychological flexibility (large effect size), well-being (medium effect size), and efficient time management (large effect size), as well as reduced levels of depression symptoms (medium effect sizes). These results are in line with past research on the effects of mindfulness on mental health and well-being (for reviews, see [70,71]), and further documented the effects of such interventions on psychological flexibility. Furthermore, the results showed an effect on increased effective time use of the participants. This sub-scale measures the absence of procrastination and of time experienced as wasted in one’s daily life. It requires self-regulation: organization and planning of activities, task initiation and execution, and ability to motivate oneself. This result is thus in line with past research underlining the relation between mindfulness and self-regulation [72], although most results reported attention or emotion regulation enhancement, and more research is needed to study other self-regulatory processes that may be useful to reduce stress and burnout at work.

Preliminary studies have shown that mindfulness influenced time perspectives. Two facets of mindfulness appeared to be most related: non-judging of experience and non-reactivity to inner experience, which were highly correlated with low PN (negative and aversive view of the past) and FN (negative views or attitudes toward the future) temporal frames, keys to establishing a balanced time perspective [49]. Two paths through which mindfulness could modify time perspectives have been proposed. The first path might be through enhancing emotion regulation, i.e., by improving one’s ability to cope with negative emotions. Being mindful may reduce the emotional reactivity when focusing on negative aspects of past/future. The second path might be through modulation of persistent negative thought processes such as rumination (for past-oriented persons) and worry (for future-oriented persons). The results of the present study suggested that a better emotional regulation and low rumination/worry through mindfulness training may improve efficient time use.

Attention switching is a complex cognitive ability, and the performance in task switching relies on three facets: switch cost (the difference in reaction times between switching and no-switching modes), no-switch cost (cost of being in a dual-task rather than a single-task mode), and processing speed [57]. In a study focusing on brief mindfulness meditation, no effect on switch cost was found, but reaction times were reduced, which suggests no-switch cost and/or processing speed were increased. A proposed mechanism to explain this result is the level of anxiety, as it is known to affect cognitive performances. Mindfulness would reduce anxiety and decrease switch costs and/or increase processing speed. Thus, our results showing both a more efficient time use and reduced anxiety are consistent with these conclusions.

The descriptive statistics of the baseline characteristics showed that the French faculty members had degraded mental health and well-being. This should encourage giving more attention to workload, time pressure, and multi-tasking in academia. Mindfulness-based programs may be a means of helping academics to decenter, reduce pressure, and take more breaks in order to prevent burnout. The present study showed that about a quarter of the members of a large department were interested in participating in a mindfulness-based intervention, which is a substantial portion. Importantly, the head of the department highly supported this initiative. University top management may find mindfulness an interesting option, as the cost of mental health problems in universities is high, and the impact of such practices may also be beneficial to students. Indeed, research has shown that mindfulness practices for teachers reduced interpersonal problems (e.g., [73]), and enhanced stress and emotion regulation competencies (e.g., [74]). Enhancing psychological and cognitive flexibility has been shown to be effective in reducing occupational stress (e.g., [31]), which was the main goal of our study. These types of programs should, however, be proposed as an option rather than becoming compulsory, as they require a high level of adherence in order to engage in daily practices and lead to positive outcomes (e.g., [75]). Indeed, recent research has shown that adherence to practices predicted a greater tendency to respond mindfully to daily events ([76]).

Although the results of our study may be promising for future research in academia, several limitations need to be underlined. The first limitation is that distribution of participants in the intervention and wait-list control groups was not random, as they could choose to take part in the mindfulness-based program immediately or later. Furthermore, both groups initially differed in several of the variables at baseline. Individuals who chose to take part in the program first showed higher levels of distress, which may have motivated them to take part in an intervention that could be helpful. Statistical analyses therefore took these initial differences into account by controlling for baseline levels. Despite this limitation, mixed ANOVAs enabled the indication of changes specific to the intervention group between t1 and t2. Various researchers working on interventional studies have claimed that randomly assigning participants to intervention and control groups may not be an optimal means of assessing the efficacy of interventions as motivation to practice represents a key element in program efficacy (e.g., [77]).

Another limitation is the sample size (41 participants) as various results were marginally significant. This study thus needs to be replicated in academic settings using larger samples in order to ensure sufficient statistical power.

## 5. Conclusions

This study indicated that mindfulness-based programs may offer interesting perspectives to improve mental health and efficient use of time in academics. Further work is needed to understand precisely how mindfulness training can improve time management. Complementary mindfulness-based interventions are also developing in various settings, including university settings, based on informal mindfulness practices, in order to be more accessible to academics who may find it too difficult to engage in 45 min practice six days per week and will not engage in such a program (for a meta-analysis, see [78]). This type of program has shown benefits on reduced stress, anxiety, and depression, as well as enhanced satisfaction with life, and these effects were mediated by increased mindfulness [79]. This suggests that it is possible to increase mindfulness through mindfulness practices integrated into everyday life such as mindful walking or mindful eating. Further studies could investigate how such practices may help academics’ efficient time management.

## Figures and Tables

**Table 1:** Socio-demographic sample description. Numbers and % in ( ) are indicated.

	Intervention Group	Control Group	The Whole Department *
All participants (N = 41)	20	21	184
Female	12 (0.6)	13 (0.62)	85 (0.46)
Male	8 (0.4)	8 (0.38)	99 (0.54)
Permanent staff	15 (0.75)	16 (0.76)	125 (0.68)
Short-term contracts	5 (0.25)	5 (0.24)	59 (0.32)
Age (years)	39.1	40.2	41.7

* permanent staff and short-term contracts employees only, interns not included.

**Table 2:** Detailed steps of the eight sessions of the intervention.

Session	Opening	Guided Practices	Between-Session Practices
1	Presentation of the instructor; presentation of general guidelines (e.g., confidentiality…); presentation of each participant and their goals Introduction about stress at work (e.g., what is stress? How does it work?)	Mindful eating experience with a raisin Body scan	Daily body scan One mindful routine (e.g., teeth brushing) One mindful meal at work or at home
2	Short introduction meditation	Body scan Discussion about between-session practices Walking down the street exercise Sitting meditation	Pleasant events calendar (write down every evening the agreeable events of the day and their effects on thoughts, emotions, and sensations) Body scan 15 min sitting meditation One new mindful routine (e.g., walking to the workplace).
3	30–40 min sitting meditation (with a focus on breath and body sensations)	Three minutes of mindful breathing (awareness, focus, and widening of attention) Mindful movements Mindful walking Calendar of unpleasant events	Sitting meditation for 15 min every other day Mindfulness movements Calendar of unpleasant events “Three minutes breathing space” three times a day
4	“See or hear” exercise (each participant chooses either to observe through the window what they see without judgment and welcoming their experience, or to listen to the current sounds)	35 min of seated breathing Feedback and discussion on home practices Discussion about stress (perception, regulation strategies) Three minutes of mindful breathing Walking mindfully in a quiet area of the room	Sitting meditation every other day Yoga or mindful walking once a day Three minutes of “facing stress”, breathing each time stress rises in the workplace
5	Opening session with three minutes of breathing space	20 min of sitting meditation incorporating visualization of a recent difficulty experienced at work (stress, tension) and reactions that emerge in the experience Feedback and discussion of practices done between sessions Three minutes of breathing space For 20 min, mindful dialogue exercise (mindful listening to a person telling a personal story of their choice) Reading of “The King and his Sons” Three minutes of mindful breathing	Sitting meditation “with difficulty” Three minutes of mindful breathing Three times a day
6	At the beginning of the session, three minutes of conscious breathing	20 min of sitting meditation with difficulty Feedback and discussion about the between-session practice; Exercise of mindful dialogue on the theme “why am I doing this job?”; exercise on mood, alternative thoughts, and perspectives when occupational stress arises Three minutes of breathing space	Sitting meditation once a day Three minutes of mindful breathing and “coping with occupational stress”
7	Opening session with a meditation called “meditation without an object” (attention is first focused on the breath, then the participant is invited to abandon any observation of thoughts, emotions, or sensations, but simply to be present)	Feedback and discussion about inter-session practices Exercise “take care of myself and act”: elaboration of a list of activities for a typical day and ways to become more mindful during activities	10 to 30 min of exercises by composing your own mindfulness program among all the practices already discussed
8	At the opening of the session, three minutes of conscious breathing	Body scan for 45 min Feedback and discussion on between-session practices Review of the whole program Discussion: how to maintain formal and informal practice after the program Three minutes of conscious breathing General feedback on the program by each participant; Closing of the program	Practices most useful for each participant

**Table 3:** Description of the online questionnaire.

Questionnaires	Nature of Collected Data	Nb Items	Scale	Justification	Cronbach’s Alpha
Personal data	Age, gender	2	-	Double-checking of t1 and t2 data pairing	
Multi-dimensional Psychological Flexibility Inventory [63]	Psychological flexibility	24	1–6	Measuring one of the mechanisms of action of mindfulness based interventions on mental health and potentially time management change	0.84
DASS-21 [64]	Depression, anxiety, and stress	21	0–3	Measuring mental health	0.82, 0.71 and 0.75
Warwick–Edinburgh-Mental Well-Being Scale [65]	Subjective and psychological well-being	14	1–5	Measuring well-being	0.87
Optimal Time Use Inventory * [66]	Time management (self-congruent time, control over one’s time, balance of activities, efficient time, optimal time use index)	25	1–5	Measuring the different facets of time management	0.82

* translated/back-translated [67,68] for this study.

**Table 4:** Comparison of the intervention and control group before intervention. Data are from pre-intervention time t1 (N = 41). A student t test to compare the means of both groups was performed. ns = non-significant; ^+^ 0.05 ≤ *p*-values < 0.1, * *p* < 0.05, ** *p* < 0.01.

	Intervention Group (N = 20)	Control Group (N = 21)
MPFI (psychological flexibility)	83.85 **	95.57
DASS-21 (stress)	17.50 ns	15.81
DASS-21 (anxiety)	7.80 ^+^	4.38
DASS-21 (depression)	13.30 ^+^	8.95
WEMWBS	43.15 *	48.09
OTUI (global)	74.45 ns	80.24
OTUI (self-congruent)	24 ^+^	26.76
OTUI (control)	15 ns	15.09
OTUI (balance)	18.85 ns	17.19
OTUI (efficient)	16.6 **	21.19

**Table 5:** Mental health scores of the studied sample. % of the participants in the different DASS-21 categories from normal (low occurrence of stress, anxiety, depression symptoms) to extremely severe (very high occurrence of stress, anxiety, depression symptoms). Data from intervention and control groups were combined (N = 41).

	Stress	Anxiety	Depression
Normal	24.4	70.7	48.8
Mild	41.5	4.9	19.5
Moderate	26.8	12.2	14.6
Severe	4.9	4.9	14.6
Extremely severe	2.4	7.3	2.4

**Table 6:** Intervention effects on psychological flexibility, mental health, well-being, and time use. Data (N = 41) were analyzed using a mixed repeated-measures ANOVA. ns = non-significant, ^+^ 0.05 ≤ *p* < 0.1, * *p* < 0.05, *** *p* < 0.001.

	t1		t2		Time * Group	
	Int. Group (N = 20)	Cont. Group (N = 21)	Int. Group (N = 20)	Cont. Group (N = 21)	Interaction	η^2^
MPFI (psych. flexibility)	83.85	95.57	106.60	95.86	25.49 ***	0.39
DASS-21 (stress)	17.50	15.81	13.10	16.29	3.65 ^+^	0.09
DASS-21 (anxiety)	7.80	4.38	4.90	4.57	3.13 ^+^	0.07
DASS-21 (depression)	13.30	8.95	7.60	8.20	3.94 *	0.09
WEMWBS (well-being)	43.15	48.09	49.85	48.52	5.82 *	0.13
OTUI (global)	74.45	80.24	80.85	80.86	3.88 ^+^	0.09
OTUI (self-congruent)	24	26.76	25.60	26.62	ns	-
OTUI (control)	15	15.09	16.25	16.09	ns	-
OTUI (balance)	18.85	17.19	20.60	17.95	ns	-
OTUI (efficient)	16.60	21.19	18.40	20.19	6.97 *	0.15
